# An age-structured model of hepatitis B viral infection highlights the potential of different therapeutic strategies

**DOI:** 10.1038/s41598-021-04022-z

**Published:** 2022-01-24

**Authors:** Farzad Fatehi, Richard J. Bingham, Peter G. Stockley, Reidun Twarock

**Affiliations:** 1 York Cross-disciplinary Centre for Systems Analysis, University of York, YO10 5GE York, UK; 2 Department of Mathematics, University of York, YO10 5DD York, UK; 3 Department of Biology, University of York, YO10 5DD York, UK; 4 Astbury Centre for Structural Molecular Biology, University of Leeds, LS2 9JT Leeds, UK

**Keywords:** Hepatitis B, Applied mathematics

## Abstract

Hepatitis B virus (HBV) is a global health threat, and its elimination by 2030 has been prioritised by the World Health Organisation. Here we present an age-structured model for the immune response to an HBV infection, which takes into account contributions from both cell-mediated and humoral immunity. The model has been validated using published patient data recorded during acute infection. It has been adapted to the scenarios of chronic infection, clearance of infection, and flare-ups via variation of the immune response parameters. The impacts of immune response exhaustion and non-infectious subviral particles on the immune response dynamics are analysed. A comparison of different treatment options in the context of this model reveals that drugs targeting aspects of the viral life cycle are more effective than exhaustion therapy, a form of therapy mitigating immune response exhaustion. Our results suggest that antiviral treatment is best started when viral load is declining rather than in a flare-up. The model suggests that a fast antibody production rate always leads to viral clearance, highlighting the promise of antibody therapies currently in clinical trials.

## Introduction

Hepatitis B virus (HBV) is a major global health problem with a high risk of causing chronic infection and death due to cirrhosis and liver cancer. Despite pioneering use of recombinant DNA technology to create a safe and cheap prophylactic vaccine against HBV^1,2^ it is not universally deployed allowing the virus to infect $\sim$1.5 million people each year^3^. These add to the very significant burden of the $\sim$296 million chronically infected individuals, that are the consequence of the failure of their immune systems to clear a primary infection^3^. Treatment of these patients relies on generic replicase inhibitors that rapidly elicit resistance and interferon to boost antiviral responses. These treatments are largely ineffective leading to $\sim$820,000 additional HBV-related deaths annually, the largest cause of liver cancer worldwide^3^. HBV infected cells produce a significant excess of non-infectious particles, such as subviral Dane particles (SVPs) that occur at $\sim$1000–100,000-fold higher concentration than the infectious virion, allowing them to act as immune system decoys by sequestering antiviral antibodies^4,5^.

HBV infected individuals that mount an adequate immune response tend to clear the infection. During the early stages of the infection the first contribution of the immune response comes from innate immunity which reduces viral spread and facilitates an adaptive immune response. Adaptive immunity is made of two components, *cell-mediated immunity* (CD4$^+$ and CD8$^+$ T cells) and *humoral immunity* (antibodies). CD4$^+$ T cells, also known as helper T cells, assist the activity of other immune cells by releasing cytokines. CD8$^+$ T cells are not only responsible for killing of infected cells but also induce the noncytolytic “cure” of such cells while antibodies neutralise virus particles and prevent infection of cells^6,7^.

Mathematical modelling provides new insights into the various aspects of viral infections and the impact of the immune response on their clearance. Ciupe *et al.* presented a model to study the role of cytolytic and noncytolitic immune responses and the time lag associated with effector cell activation and expansion during an acute HBV infection. It is hypothesised that cured cells and their progeny are less likely to get infected, preventing reinfection^8,9^. Later, Ciupe *et al.* looked into the dynamics of antibodies and showed that retaining a strong cell-mediated immune response is crucial for the control of early infection in unvaccinated individuals^5^. Fatehi *et al.* developed a mathematical model to take into account contributions from innate and adaptive immune responses, as well as cytokines. The model investigates the roles of different components of the immune response on viral dynamics^10^. These models are based on systems of ordinary differential equations (ODE) or delay differential equations (DDE). Nelson *et al.* presented an age-structured model of human immunodeficiency virus (HIV) infection to study the impact of variations in the virion production rate and the death rate of infected cells over the course of the infection^11^. Although age-structured models are more complicated, they can provide more realistic dynamics^12^.

Experimental studies have shown that persistent stimulation of effector cells may result in immune impairment, e.g., immune exhaustion^13–15^. In HBV infections, persistent antigen presentation by infected cells and exposure to high antigen loads plays an important role in CD8$^+$ T cell exhaustion^16^. In order to analyse the impact of T cell exhaustion on viral dynamics, Johnson *et al.* introduced a variable that captures the antigenic stimulus, called the level of exhaustion^17^. They assumed that T cells are inactivated dependent on the level of exhaustion and modelled it as a Hill function with a half-maximal constant called the exhaustion threshold. They showed that the exhaustion threshold has a significant impact on the ability of the immune response to control an infection^17^. Later, Conway and Perelson included T cell exhaustion into an HIV infection model and showed that the strength of cytotoxic T lymphocytes in killing productively infected cells, and the level of latently infected cells, determine the post-treatment outcome of the infection^18^.

We recently introduced a model of intracellular HBV infection dynamics and used it for comparative analysis of different therapeutic strategies^19^. The model reveals a two-phase behaviour in the release of non-infectious SVPs. Shortly after infection, a cell starts secreting SVPs. When the first intact virions are released, after $\sim$90 hours post-infection, SVP secretion stalls until the onset of a second secretion phase. In order to analyse the impacts of this behavior on the immune response dynamics, and in particular their interaction with anti-HBsAg antibodies, we have developed an age-structured model using infection kinetic parameters derived from our intracellular model. This model has been parameterised such that its outputs fit data recorded from patients undergoing an acute infection. We have adapted to the scenarios of a chronic infection, immune clearance of the infection, and infection flare-ups via variation of the immune response parameters. The role(s) of cell-mediated and humoral immunity in an acute HBV infection, including the effects of immune response exhaustion, have been analysed. The impacts of direct-acting antivirals (DAA) treatments targeting various steps of the virus life cycle are compared to blocking T cell exhaustion, which is known as exhaustion therapy. Optimal treatment regimes, such as the most effective treatment start times for the reduction of viral load, have been identified. Our analysis highlights the potential benefit of antibody therapies currently in clinical trials, and allows modelling of the impacts of DAAs under development.

## Results

### Model derivation

In order to analyse HBV infection dynamics with emphasis on  the roles of the immune response, we developed a detailed intercellular age-structured model of HBV infection. This model includes uninfected target cells, *T*(*t*), which are assumed to be created at rate $\lambda$ and to die at a rate *d*^18,20^. Each individual infected cell is characterised by its age, *a*, since infection. Therefore infected cells are denoted as *I*(*a*, *t*)^11^. An infected cell produces complete virions, which are infectious, and incomplete particles which occur in three distinct forms: RNA containing particles; empty virions; and subviral particles (SVPs) which are either filamentous or spherical^4^. The release dynamics of these particles from an infected cell has been studied precisely^19^. The variable $V_c(t)$ indicates the number of complete virions which infect target cells at a rate $\beta$. All types of incomplete particles are non-infectious and present HBV surface antigens (HBsAg), enabling them to act as decoys for the immune response by depleting HBsAg-specific antibodies, *A*(*t*). We therefore only include the variable $V_i(t)$ into the model, which represents the total number of incomplete particles^4^. The production rate of these particles depends on the age of an infected cell. The functions $P_c(a)$ and $P_i(a)$ show the production profiles of complete and incomplete particles, respectively, from infected cells of age *a*^11^. We use the functions that are presented in Fig. 2c of^19^ as the base functions $P_c(a)$ and $P_i(a)$ (see Supplementary Fig. S1 online). Since it has been shown that changing the intracellular model parameters will change the total number of released particles, we scale functions $P_c(a)$ and $P_i(a)$ by $\rho _1$ and $\rho _2$, respectively. We assume that $\rho _1$ and $\rho _2$ are changeable parameters and fit them to patient data. Complete and incomplete particles are cleared, based on their half-life, at rates $d_c$ and $d_i$. HBsAg-specific antibodies, which are a component of the adaptive immune response called *humoral immunity*, are produced at rate $p_A$ proportional to antigen load, and are degraded at rate $d_A$. We also add a logistic growth to the antibody equation, which is maintained through homeostatic proliferation of memory B cells after infection. Antibodies can bind to complete and incomplete particles at rate $k_f$ to create complete virion-antibody complexes, $X_c$, and incomplete particle-antibody complexes, $X_i$, respectively. These complexes disassociate at rate $k_b$, or degrade at rate $d_x$^5^.

The other component of an adaptive immune response is *cell-mediated immunity*. This occurs via the action of CD8$^+$ T cells, also referred to as effector cells, *E*(*t*). These cells kill or cure infected cells. Since it has been hypothesised that cured cells lose their resistance to productive infection at a slow rate of the order of $10^{-5}$ per day^8^, we model these two impacts in one reaction, where effector cells remove infected cells at rate $\mu$. Effector cells target infected cells in response to the display of viral peptide-major histocompatibility complex (MHC) on the cell surface, which is dependent on the expression of viral proteins in the cells. As the intracellular model has shown, these viral proteins are produced and secreted shortly after infection. We therefore assume that the removal rate of the infected cells is age-independent^19^. In addition, we assume infected cells die at rate $\delta$ due to infection or the action of the innate immune response, which is not included in the model directly^5,10^. Effector cells are assumed to be produced at rate $\lambda _E$ and removed at rate $d_E$ in the absence of infection^8^. During infection, proliferation of effector cells happens in an infected cell density dependent manner with a time delay, i.e. it depends on the density of antigen, with maximum proliferation rate $\alpha$, and on $\phi$, the level of infected cells at which proliferation is half-maximal^8,17,21^. It has been argued that persistent exposure to high antigen loads is associated with CD8$^+$ T cell exhaustion^16^. Therefore, functional effector cells are lost at maximal rate $\xi$, depending on the level of exhaustion, *Q*, which is implemented as a Hill function with coefficient *n* and half-maximal constant $q_c$^17^. The level of exhaustion, *Q*, is measured by integrating over the antigenic stimulus (${Y}/{(\phi +Y)}$) times the parameter $\kappa$ which is called “blockade parameter”, and reduces exponentially with coefficient $d_q$, which is the rate of immune response recovery from exhaustion. The reduction of the blockade parameter is assumed to simulate the blockade of interaction between the inhibitory receptor programmed death 1 (PD-1) and its ligand (PD-L1) which restores CD8$^+$ T cell function^22^.

**Figure 1:**
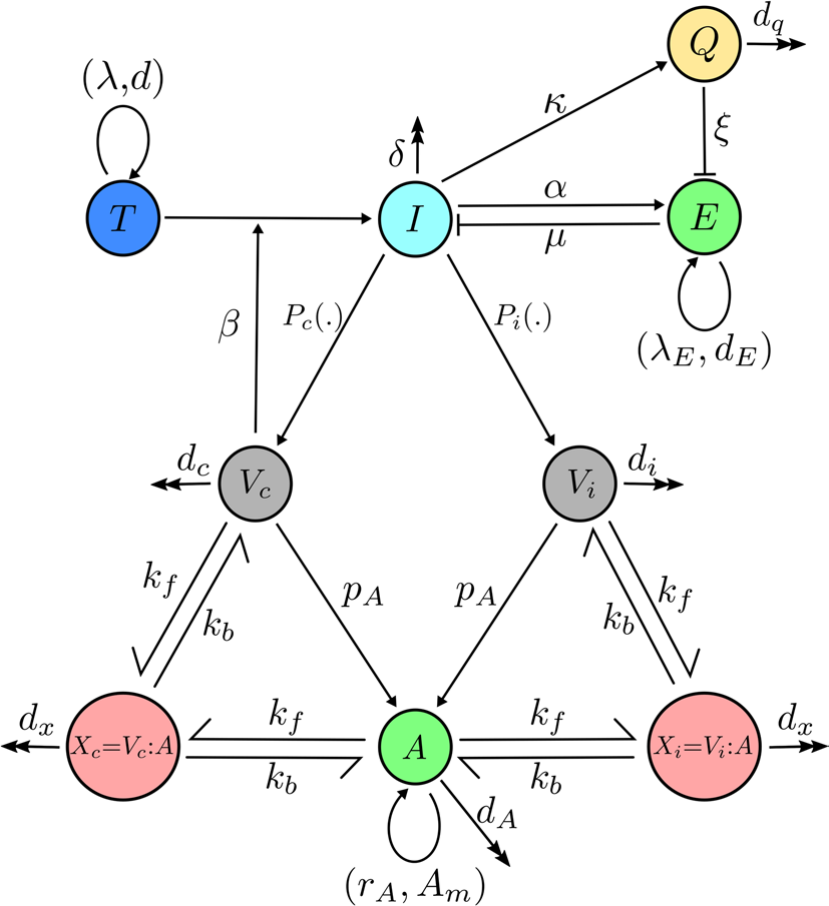
The components and inferred interactions of the age-structured model of the immune response to an HBV infection (1), enabling comparison of different treatment options. Blue and cyan circles show uninfected (*T*) and infected (*I*) cells, respectively, green circles indicate the immune response (antibodies (*A*) and effector cells (*E*)), and gray circles show complete ($V_c$) and incomplete ($V_i$) viral particles. Pink circles represent complete virion-antibody ($X_c$) and incomplete particle-antibody ($X_i$) complexes. Yellow shows the level of exhaustion (*Q*). Double arrow-headed lines indicate natural clearance, bar-headed and single arrow-headed lines indicate destruction and production/proliferation, and forward/backward arrows represent binding/unbinding events.

With the above assumptions, the model, as illustrated in Fig. 1, takes on the form:

1 $$\begin{aligned} &\displaystyle {\dfrac{dT}{dt}=\lambda - d T-\beta TV_c,}\\&\displaystyle {\dfrac{\partial I(a,t)}{\partial t}+\dfrac{\partial I(a,t)}{\partial a}=-\delta I(a,t)-\mu I(a,t)E,}\\&\displaystyle {\dfrac{dV_c}{dt}=\rho _1\int \limits _0^{\infty }P_{c}(a)I(a,t)da-d_{c}V_c-k_fAV_c+k_bX_{c},}\\&\displaystyle {\dfrac{dV_i}{dt}=\rho _2\int \limits _0^{\infty }P_{i}(a)I(a,t)da-d_{i}V_i-k_fAV_i+k_bX_{i},}\\&\displaystyle {\dfrac{dA}{dt}=p_A(V_c+V_i)+r_AA\left( 1-\dfrac{A}{A_m}\right) -d_AA-k_fA(V_c+V_i)}\\& \qquad \displaystyle {+k_b(X_c+X_i),} \end{aligned} \quad \quad \begin{aligned}&\displaystyle {\dfrac{dX_c}{dt}=k_fAV_c-k_bX_{c}-d_xX_c,}\\&\displaystyle {\dfrac{dX_i}{dt}=k_fAV_i-k_bX_{i}-d_xX_i,}\\&\displaystyle {\dfrac{dE}{dt}=\lambda _E+\alpha \dfrac{Y(t-\tau )}{\phi +Y(t-\tau )}E(t-\tau )-\xi \dfrac{Q^n}{{q_c}^n+Q^n}E}\\& \qquad \displaystyle {-d_EE,}\\&\displaystyle {\dfrac{dQ}{dt}=\kappa \dfrac{Y}{\phi +Y}-d_qQ,}\\&\\&\\& \end{aligned}$$

where $$\displaystyle {I(0,t)=\beta TV_c}$$ and $$\displaystyle {Y=\int \limits _0^{\infty }I(a,t)da}$$.

All parameter descriptions and values adapted from the literature are presented in Table 1 together with a pointer to the references they have been adapted from. The remaining parameters are estimated by fitting the model (Fig. 1).

**Table 1:** Overview of model parameters. Parameters that are fixed in the model are either adapted from the references indicated, or are fitted.

Parameter	Description	Value	Reference
$\alpha$	Effector cell growth rate	$\text{ ml } \text{ day}^{-1}$ (varies)	–
$A_m$	Antibody carrying capacity	$3.4\times 10^{12} \text{ per } \text{ ml }$	^5,26^
$\beta$	Infection rate	$\text{ ml } \text{ day}^{-1}$ (varies)	–
$\delta$	Infected cells death rate	$\text{ day}^{-1}$ (varies)	–
*d*	Death rate of target cells	$0.01 \text{ day}^{-1}$	^20,25^
$d_A$	Antibody degradation rate	0.033 $\text{ day}^{-1}$	^5^
$d_c$	Virus clearance rate	0.67 $\text{ day}^{-1}$	^5^
$d_E$	Removal rate of effector cells	0.5 $\text{ day}^{-1}$	^9^
$d_i$	Incomplete particle clearance rate	0.67 $\text{ day}^{-1}$	^5^
$d_q$	Reduction rate of exhaustion level	0.1 $\text{ day}^{-1}$	^17^
$d_x$	Complex degradation rate	2.7 $\text{ day}^{-1}$	^5^
$\kappa$	Growth rate of exhaustion level	1 $\text{ day}^{-1}$	^17^
$k_b$	Antibody dissociation rate	10 $\text{ day}^{-1}$	^5^
$k_f$	Antibody binding rate	$10^{-10} \text{ ml }\text{ day}^{-1}$	^5^
$\lambda$	Target cells production rate	$4\times 10^5 \text{ per } \text{ ml }$	^8,23,24^
$\lambda _E$	Effector cell production rate	10 $\text{ per } \text{ ml }$	^9^
$\mu$	Removal rate of infected cells by effector cells	$\text{ ml } \text{ day}^{-1}$ (varies)	–
*n*	Hill function coefficient for effector cell exhaustion	3	^17^
$\phi$	Hill function half-maximal constant for effector cell growth	$10^6 \text{ per } \text{ ml }$	Fitting
$p_A$	Antibody production rate	$\text{ molecule } \text{ day}^{-1}$ (varies)	–
$q_c$	Hill function half-maximal constant for effector cell exhaustion	10	^17^
$\rho _1$	Rate modifying for virus production rate	varies	–
$\rho _2$	Rate modifying for incomplete particle production rate	varies	–
$r_A$	Antibody proliferation rate	$\text{ day}^{-1}$ (varies)	–
$\tau$	Effector cell growth time delay	$\text{ day }$ (varies)	–
$\xi$	Effector cell maximal exhaustion rate	$1 \text{ day}^{-1}$	Fitting

### Within-host viral dynamics

**Figure 2:**
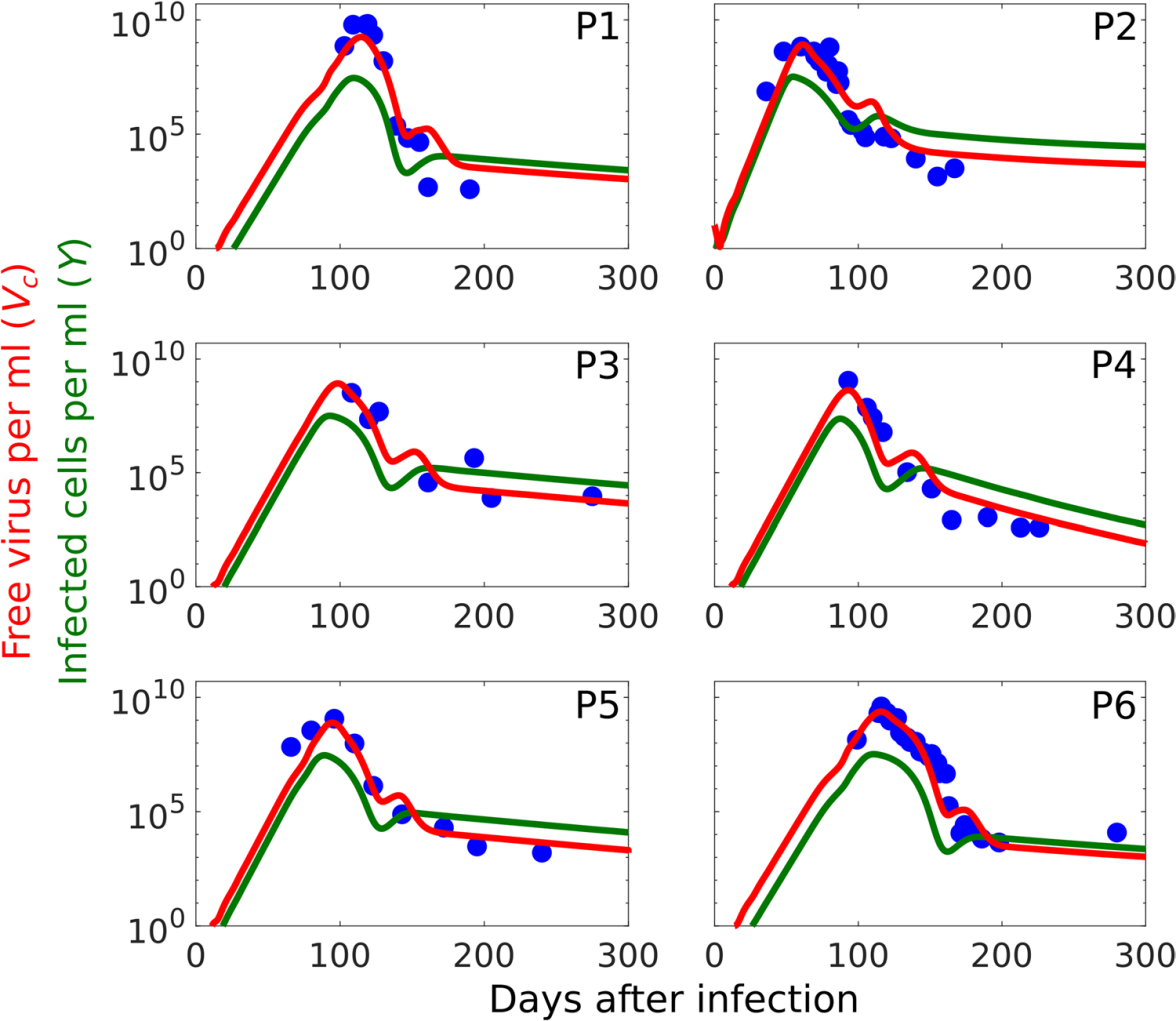
Paramaterising the model against patient data. The fitting of $V_c$ as given by model (Fig. 1) (red line) to patient data ($\bullet$) indicates a biphasic decline in viral load and sometimes a slower decline in the level of infected cells compared with virions (green line).

The model (Fig. 1) was fitted to viral load data from 6 patients who were recorded during the acute stage of infection (Fig. 2)^5,8^. In this work, we also let the initial viral load ($V_c(0)$) be a variable. In patient number 2 (Fig. 2 P2) the peak in viral load occurs earlier than in other patients, which shows the initial viral load could be higher in this patient. We observe that for patient numbers 1, 3, 4, 5 and 6, $V_c(0)=0.33$ virion per ml as suggested by Ciupe *et al.*^5^, but for patient number 2 $V_c(0)=10$ virions per ml. The parameter values derived from fitting of $V_c$ are presented in Table 2. Our model captures the essential features of viral loads in all patients, including the first rapid decline in the level of viral load after the peak, followed by a slower decline, i.e. the biphasic viral decay after the peak^8^. In all patients viral load eventually decreases to zero, matching the clinical outcomes in these patients with acute HBV infections.

The average fraction of infected cells at the peak of infection is around 74.5%, with a range of 60%-82%. This is in good agreement with previously estimated values^8,9^, and the experimental observation that more than 75% of hepatocytes were hepatitis B core protein antigen positive ($\text{ HBcAg}^+$) in infected chimpanzees^6^. Previous models suggested that the level of infected cells has a biphasic decline and decreases faster than the viral load^8,9^. However, it has been observed that the level of cccDNA (infected cells) has a slower decline compared with the level of free virus and remains detectable for more than two years after infection^27^. Our model predicts that infected cells decline slower compared with viral load (green lines Fig. 2), in good agreement with Fig. 2 in Wieland *et al.*^27^. We fitted parameter values from Table 2 to a Gaussian distribution, inferred 100 parameter sets and plotted the outcome for the 100 inferred parameter sets (see Supplementary Fig. S2 online). Consistent with the fact that these patients are acute cases, we observe acute infection in 60% of the simulations, a periodic solution in 25% of simulations, while the remainder exhibit a stable chronic infection steady state with a low level of free viruses (around $10^5$ virions per ml).

**Table 2:** Parameter best estimates

Patient	$\beta \times 10^{-9}$	$\mu \times 10^{-4}$	$\delta$	$\rho _1$	$\rho _2$	$p_A\times 10^{-4}$	$r_A$	$\alpha$	$\tau$	$V_c(0)$	RSS
1	0.35	1.00	0.0130	8.50	2.50	3.00	0.366	2.20	6.50	0.33	0.29
2	3.57	2.00	0.0210	3 .00	0.70	1.00	0.550	1.50	4.50	10.00	0.14
3	1.20	0.90	0.0170	3.20	0.70	1.00	0.391	1.80	5.50	0.33	0.29
4	1.70	2.00	0.0400	3.00	0.70	1.00	0.370	1.80	5.00	0.33	0.24
5	1.30	1.80	0.0180	3.50	0.70	1.00	0.430	1.80	6.00	0.33	0.22
6	0.35	1.00	0.0130	8.50	2.50	3.00	0.366	2.20	8.70	0.33	0.13
Median	1.25	1.40	0.0175	3.35	0.70	1.00	0.381	1.80	5.75	0.33	–
Mean	1.41	1.45	0.0203	4.95	1.30	1.67	0.412	1.88	6.03	1.94	–
Std	1.19	0.54	0.0101	2.76	0.93	1.03	0.072	0.27	1.49	3.95	–

### Adaptive immune response dynamics

**Figure 3:**
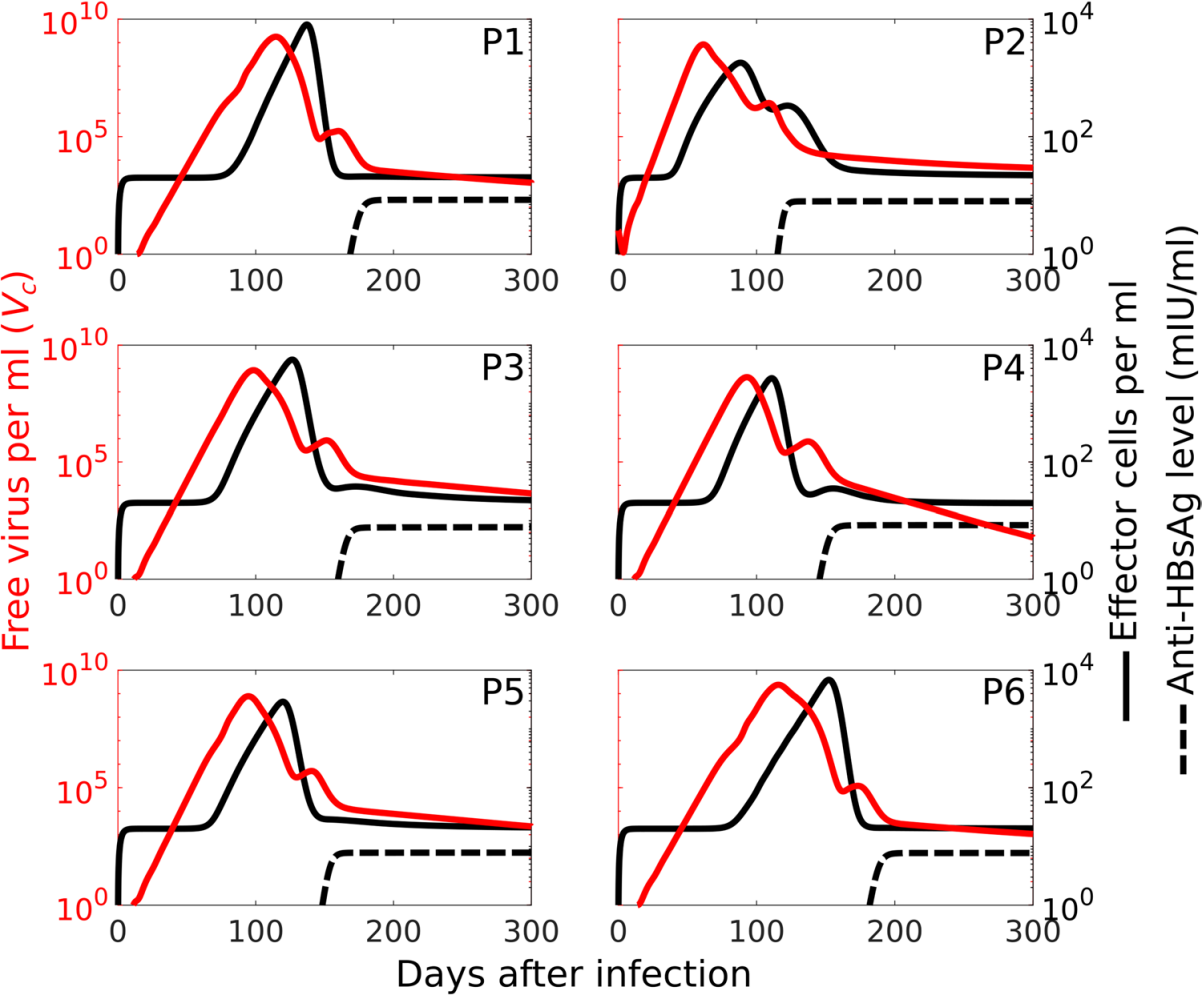
Time course of the immune response to the HBV infection in terms of effector cell and anti-HBsAg levels. The maximum in effector cells (black curve) occurs 3–4 weeks after the peak in viral load. The red lines show the best fit to patient data, and the black dashed lines show the level of anti-HBsAg in mIU/ml unit as predicted by the model (Fig. 1).

**Figure 4:**
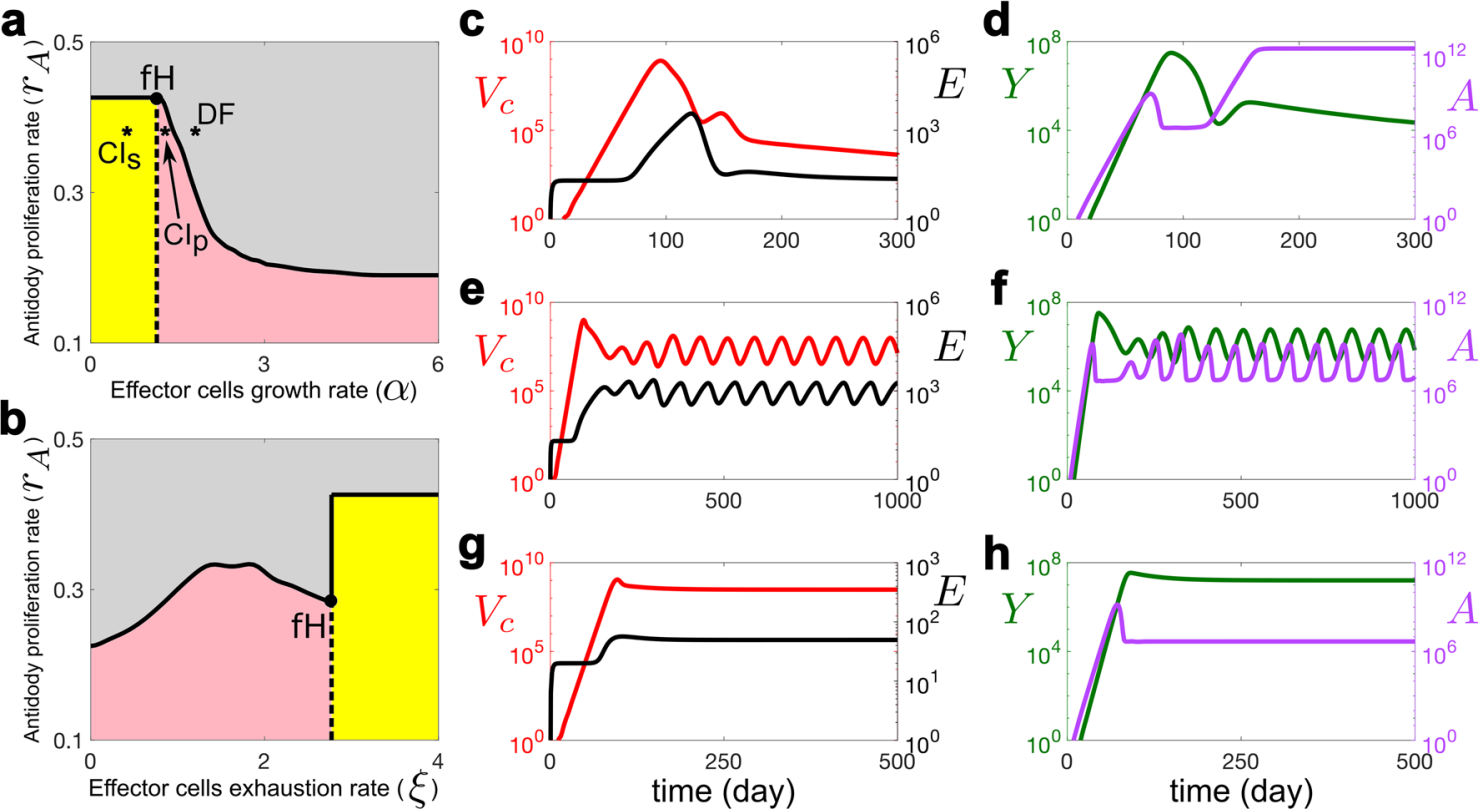
Stability analysis of the disease-free and chronic infection states, exemplified for different types of infection dynamics: acute infections with immune clearance, and chronic infections with and without infection flare-ups. The parameters used are the median values from Table 2. The gray and yellow areas in (**a**) and (**b**) indicate the regions where the disease-free (DF) and chronic infection (CI_s_) steady states are stable, respectively. Pink is the region where the system shows a stable periodic solution around a chronic infection steady state (CI_p_). Solid and dashed lines indicate the boundaries of the steady-state and Hopf bifurcation, respectively, and “fH” pinpoints the location of the fold-Hopf bifurcation. Stars indicate the points that are used for model simulations. (**c**) and (**d**) correspond to an acute infection. $\alpha$, the maximum proliferation rate of effector cells, in (**e**) and (**f**) is reduced by 28% ($\alpha =1.3$), indicating periodic oscillations around the chronic infection steady state (hepatitis flare). In (**g**) and (**h**), $\alpha$ is reduced by 56% ($\alpha =0.8$), illustrating the case of a chronic infection.

The level of serum alanine aminotransferase (ALT) is used as proxy for the dynamics of effector cells in HBV infection^8,9^. It has been observed clinically that the peak in serum ALT occurs after the peak in viral load and the time lag between these two peaks is 3–4 weeks^8,28^. Our model also predicts that the average time lag in these 6 patients is 26.18 days (Fig. 3) which is in good agreement with previously reported values^8,28^. We observe this delay in all patients (Fig. 3) and in our model (Fig. 4c). This is due to the time lag in the production and activation of the effector cells and their propagation from lymph node to the tissue. This delay creates a window of therapeutic  opportunity to start treatment with maximal effect while viral load is decreasing and effector cells are approaching their maximal value. To provide a more realistic dynamic of effector cells we included their exhaustion into our model. Supplementary Fig. S3 online indicates that in each patient the onset of exhaustion coincides with a step increase in the level of effector cells. However, before the peak in exhaustion level, the immune response controls the infection, so that the level of exhaustion starts declining before the peak of effector cells is reached.

Figure 3 indicates the level of antibodies against HBsAg (anti-HBsAg) in these patients. Anti-HBsAg levels $<10 \text{ mIU/ml }$ ($8.5\times 10^{-4} \text{ mg/ml }$) are considered as negative^5,29^. In unvaccinated patients, the level of antibodies is under 10 mIU/ml while they are still classed as infected, i.e. their HBV-DNA test is positive^26^. Figure 3 shows that in all patients the level of antibodies is well below 10 mIU/ml during the peak in viral load and the first phase of viral decline. Later, when the level of virus has decreased significantly and effector cells are reduced to their basal level, it starts increasing but is still under 10 mIU/ml^26^.

Now we study the impact of varying immune response parameters on viral dynamics. We focus on parameters $\alpha$ (growth rate of effector cells) and $\xi$ (the rate at which functional effector cells are lost due to the exhaustion) in the cell-mediated immune response which play crucial roles in the control of the infection^10,28^. The other important parameter is the antibody proliferation rate ($r_A$)^5^. Figure 4 shows the stability and instability regions of the disease-free and chronic infection states as a function of these parameters. This figure shows that an increase in $r_A$ always stabilises the disease-free state. However, when $r_A$ is low ($<0.22$), increasing $\alpha$ or decreasing $\xi$ cannot stabilise the disease-free state, However, it destabilises the chronic steady state due to a Hopf bifurcation, so one observes stable oscillations. Biologically, this is known as “flare-up”, where through the interactions between viral infection and immune response we observe a periodic behaviour^10,30,31^. Examples of different types of infection dynamics are shown in Fig. 4 where Fig. 4c and d indicate acute infection, Fig. 4e and f show a chronic infection with flare-ups, and Fig. 4g and h show a chronic infection without flare-ups (stable chronic infection steady state). Supplementary Fig. S4 online illustrates the impact of the time delay in effector cell activity ($\tau$) and of the removal rate of infected cells by effector cells ($\mu$) on viral dynamics. Supplementary Figs. S4a and b online indicate that a decrease in $\tau$ increases the stability range of the disease-free state with respect to $r_A$ and $\xi$, respectively. That is, following a decrease in $\tau$, the disease-free steady state will become stable for lower values of $r_A$ and higher values of $\xi$. Supplementary Fig. S4c online shows a similar effect with respect to $\mu$. This figure shows that an increase in $\mu$ results in an increase in the stability range of the disease-free state.

### Effects of current clinical therapies

There are currently two approved anti-HBV therapies for clinical use. Interferon (IFN)-based therapy and nucleot(s)ide analogues (NAs)^32^, but more therapeutic strategies will be required to meet the Global WHO Challenge to make HBV infection a treatable condition by 2030^16^. We recently compared current DAAs with several possible future treatment options using our intracellular model^19^. The latter include capsid assembly modulators (CAMs), especially based on heteroaryldihydropyrimidines HAPs^33^. Recently we showed that both HCV and HBV regulate the assembly of their nucleocapsids around ssRNA forms of their genomes (e.g. the pgRNA of HBV) at least in part via sequence-specific core protein-genomic RNA interactions at multiple sites, termed Packaging Signals (PSs)^34–36^. CAMs are at an advanced stage of development^37–39^, whilst the vital roles of PS-core interactions in HBV nucleocapsid assembly, and their targeting by small ligands are at a much earlier stage. The comparative analysis reveals strong potential synergistic effects between them. These treatment options reduce the viral production rate and their effect is modelled by a factor $(1-\varepsilon _1)$, where $0\le \varepsilon _1\le 1$ is the drug efficacy and its value is constant from the start of the treatment until its removal^20,40^. This method has also been used in the context of other viruses^41–45^. IFN-based therapy and CAMs also block *de novo* infections^10,46^. Mathematically, one can represent this effect by a modified transmission rate $(1-\varepsilon _2)\beta$, where $0\le \varepsilon _2\le 1$ is the drug efficacy^10,19^. Here we refer to these types of treatments as antiviral therapy. The other suggested form of therapy is the recovery of the CD8$^+$ T cells from exhaustion (exhaustion therapy)^16^. The impact of this treatment option can be modelled by a modified blockade parameter $(1-\eta )\kappa$, where $0\le \eta \le 1$ is the efficacy of the treatment^22^. The new equations take the following form:

2 $$\begin{aligned} \begin{array}{l} \displaystyle {\dfrac{dT}{dt}=\lambda - d T-(1-\varepsilon _2)\beta TV_c,}\\ \displaystyle {\dfrac{dV_c}{dt}=\rho _1(1-\varepsilon _1)\int \limits _0^{\infty }P_{c}(a)I(a,t)da-d_{c}V_c-k_fAV_c+k_bX_{c},}\\ \displaystyle {\dfrac{dQ}{dt}=\kappa (1-\eta )\dfrac{Y}{\phi +Y}-d_qQ.} \end{array} \end{aligned}$$

The total drug effectiveness $\varepsilon _{tot}$, defined as $\varepsilon _{tot}=1-(1-\varepsilon _1)(1-\varepsilon _2)$, allows us to determine a critical drug efficacy, $\varepsilon _c$, where the infection gets cleared for $\varepsilon _{tot}>\varepsilon _c$^10,47^.

**Figure 5:**
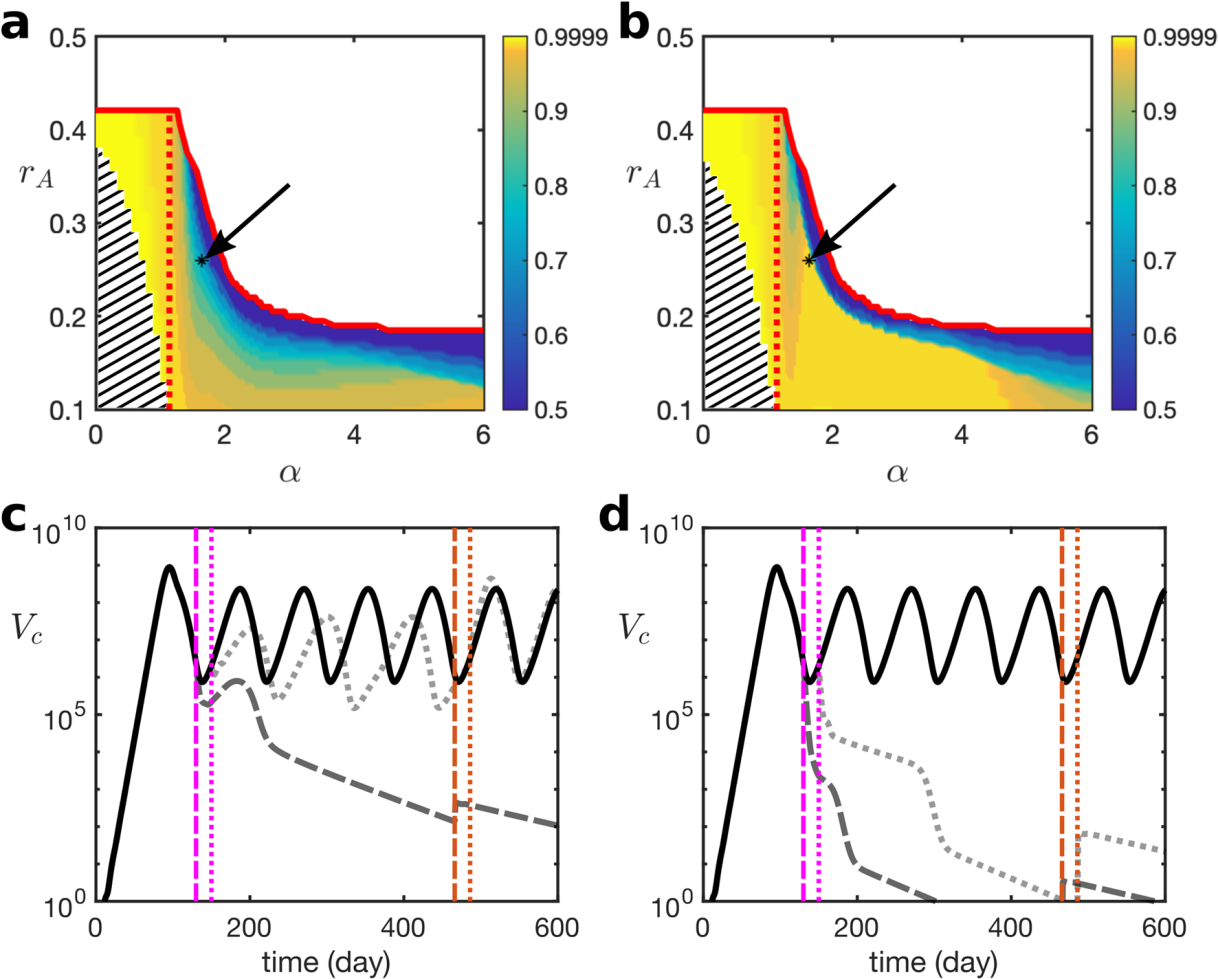
Start of antiviral therapy when viral load is in a declining phase is more effective. (**a**) and (**b**) show the minimal total efficacy ($\varepsilon _{tot}$) that is required to clear the infection following 48-weeks of therapy starting at 130 and 150 days post infection (dpi), respectively. The white area indicates a stable disease-free state. The red solid curves indicate the onset of the steady-state bifurcation, whereas the red dotted lines of the Hopf bifurcation of the chronic state. The black hatched area indicates the region where an efficacy of $\le 99.99\%$ is ineffective. The black star (indicated with an arrow) shows the point at which the simulations ((**c**) $\varepsilon _{tot}=0.7$ and (**d**) $\varepsilon _{tot}=0.99$) have been performed. Magenta and brown vertical lines indicate the start and end of treatment, respectively. The dashed and dotted lines, represent the effects of treatments on viral load, for treatment starting 130 or 150 dpi, respectively.

IFN-based therapy, which results in higher rates of hepatitis B e antigen (HBeAg) and HBsAg loss compared with NA therapy, is usually administered for around 48 weeks^32^. Thus, 48 weeks after the start of treatment we remove its effect on the model to determine the efficacy required to avoid a viral rebound after the removal of treatment. This will help us to find an optimal efficacy that a new therapy should have to be effective, thus addressing an urgent need identified by Lok *et al.*^32^. Figure 5 shows the impact of starting antiviral therapy at different times post infection. In Fig. 5a treatment start is 130 dpi, while in Fig. 5b it is 150 dpi. These figures indicate that in a region where the system shows a periodic behaviour, an earlier treatment start during the declining phase of the viral dynamics is more effective than a treatment start during the increasing phase or around the peak of a flare-up (Figs. 5c and d, verified explicitly for treatment start one week before, and after, and the top of the peak). This indicates the co-existence of the disease-free steady state and stable periodic oscillations around the chronic infection steady state, so that depending on the initial conditions the system can either converge to the disease-free case or exhibit a periodic behaviour. Thus, starting treatment at different times can lead to different outcomes. Supplementary Video S1 online shows the minimal total efficacy ($\varepsilon _{tot}$) that is required to clear the infection following 48-weeks of therapy starting at various times between 50 dpi (representing an early detection of the infection) to 160 dpi (representing a late detection of the infection). It indicates that treatment start at 50-80 dpi (around a month before the first viral peak) is most effective. It also shows that treatment start at 90-120 dpi (close to the first viral peak) is not that effective, in particular when the chronic steady state is stable. After the first viral peak, if the chronic steady state is stable, the treatment start time is not important as the infection is in a stable state. However, in the case of a periodic solution, starting treatment when the viral load is declining requires a treatment of lower efficacy than would be necessary to achieve the same outcome for a treatment start in the increasing phase. These results indicate that a decline in the level of free virus in a patient, can be a sign of an acute infection, or correspond to the declining phase before a flare-up. Therefore, it is crucial to start the treatment, rather than wait to start it after the flare-up. Supplementary Video S2 online shows the minimal efficacy required to clear the infection following 48 weeks of exhaustion therapy starting at in between 50 dpi and 160 dpi. Surprisingly, it shows that this treatment is not as effective as antiviral therapy targeting the viral life cycle and the treatment start point has no significant impact on the outcome. Moreover, our model does not show any significant synergistic effects between these two treatment options. This can be seen from the fact that this treatment option is only effective in a small area of the $r_A$-$\alpha$ parameter plane, corresponding to an area in which antiviral therapy targeting the viral life cycle is also effective even for low drug efficacy.

In Fig. 5 we consider a constant treatment efficacy for the full duration of the treatment, as is typically done in viral infection models^20,40–45^. However, in Supplementary Figs. S5 and S6 online we study the impact of a periodic administration of antiviral treatments of different intensities (efficacies), including also the option of zero efficacy to model the absence of treatment in one of the treatment periods, to find possible synergistic effects for alternating treatments. We consider treatment starts at 130 and 150 dpi, respectively, and different combinations of treatment periods, i.e. a treatment with total efficacy $\varepsilon _1$ in period 1 is followed by a treatment with total efficacy $\varepsilon _2$. Then this scheme is repeated for 48 weeks, which is the recommended time for administration of an HBV therapy^32^. Supplementary Fig. S5 online indicates that alternating antiviral therapy with a total efficacy of 90% for 4 weeks, with 4-week intervals of no treatment over 48 weeks leads to clearance of the infection. To understand the dynamical reason for this, we checked viral load 28 days post treatment stop. We observed that it is below the level of virus in the drug-free (control) case at 186 dpi (130+56) and is in a declining phase. Although for treatment start at 150 dpi the results are more chaotic (see Supplementary Fig. S6 online), cases leading to clearance of the infection show similar behaviour as for the 130 dpi case. Supplementary Fig. S7 online shows the impact of a treatment start at 150 dpi, with periodic administration of antiviral treatments of different efficacy in 4 weeks intervals. This figure shows that in successful cases antiviral treatment is administered during the declining phase of viral load. This is consistent with the main conclusion from our modelling of constant treatment efficacies above, that also shows that the outcome of a treatment depends highly on a start time in the declining phase of viral load. Although Supplementary Figs. S6 and S7 online indicate that drug administration in various efficacies and periods can sometimes lead to clearance of the infection, establishing these time periods and efficacies in a patient is not practicable in the clinic. To address this issue, we studied the impact of a treatment start at each day in one period of the viral load dynamics (i.e. each day from 130 to 212 dpi, covering 83 possible start times). In each case, we either consider a constant efficacy, or a periodic efficacy where the treatment is administered for 28 days and then withheld for 28 days. We chose an 28-day periodicity as this is optimal according to Supplementary Figs. S5 and S6 online. This shows that for a total treatment efficacy of 99% a constant treatment will always leads to viral clearance. However, if the total efficacy is only 90% then a constant treatment is successful only at 52 treatment start times i.e., the chance of having a successful outcome is 62.65%. By contrast, a periodic treatment would increase the chance of success to 96.38%. It is worth noting that all currently available treatments for HCV have an efficacy of more than 99%^20^. Also CAMs and the PS-targeting drugs for HBV, which are under development, have shown promising outcomes with efficacies of more than 95%^19^. Thus, our analysis suggests that a non-periodic treatment option, in line with those previously administered for HBV and other viral infections, appears to be a reasonable strategy. However, different strategies should be tested in clinical trials, as our modelling indicates that under certain circumstances periodic treatment options can be superior.

Monoclonal anti-HBsAg antibody drugs are currently being investigated in clinical trials as a treatment against HBV infections^48–50^. To study the impact of this treatment option we extended our model, modelling these antibodies as an influx into the system (see Supplementary Section S1 online). Supplementary Fig. S8 online indicates that the impact of monoclonal antibody therapy is independent of the treatment start time, leading to clearance of the infection in both the declining and the increasing phase.

## Discussion

In this paper we derive an age-structured model for the immune response to HBV infections. It is based on the particle release profiles from a detailed intracellular model^19^. Our age-structured model focuses on two components of the adaptive immune response known as humoral (antibodies) and cell-mediated (CD8$^+$ T cells) immunity. To provide more realistic viral dynamics and robust predictions, we fitted our model to patient data. Patient data indicate a biphasic decrease in viral load which is well captured by the model. Previous models have predicted that the number of infected cells declines faster than viral load^8,9^, but it has been shown clinically that the level of cccDNA (infected cells) has a slower decay compared with viral load^27^. Interestingly, our model reflects this trend. Regarding the dynamics of the immune response, our model shows that the peak in the level of effector cells occurs around 26 days after the peak in viral load, which is in good agreement with previously reported values^8,28^. The antibody level stays well under 10 mIU/ml during the peak and the first phase of viral decline. Later, it starts increasing, but it remains under 10 mIU/ml^26^.

We also performed a stability analysis of the model to study the impact of varying parameter values on the outcome of the infection. The model suggests that increasing $r_A$ (the proliferation rate of antibodies) always stabilises the disease-free state. When $r_A$ is low, decreasing $\xi$ (the rate at which functional effector cells are lost due to exhaustion) or increasing $\alpha$ (expansion rate of effector cells) will not stabilise the disease-free state, but the chronic steady state loses its stability and one observes stable oscillations^10,30,31^. Therefore, our model illustrates the importance of the *humoral immunity* in viral clearance.

Current therapeutic strategies either block the formation of new virions and viral entry (antiviral therapy) or recover the exhausted CD8$^+$ cells (exhaustion therapy). We used our model to compare the impacts of these different treatment options. Our results suggest that exhaustion therapy is not as effective as antiviral therapy, and that the start of treatment does not have a significant impact on the outcome of exhaustion therapy. However, the timing of treatment start plays a crucial role in the outcome of antiviral therapy. Our model shows that around one month before the peak in viral load would be the best time to start antiviral therapy, illustrating the importance of identifying cases early into an infection. Since this is not always practicable, we also analysed the impact of a treatment start later during infection. Whilst treatment start time is not significant in the context of stable chronic steady states, it matters for periodic solutions, where a treatment start during the declining phase of viral load is more effective. As declining levels of free virus in a patient can indicate the declining phase before a flare-up, it is crucial to start treatment immediately rather than wait until after a flare-up. Our model moreover illustrates that antibodies play an important role in suppression of viral load, supporting strategies to use monoclonal antibodies as therapy against chronic Hepatitis B viral infections^48–50^, and our model suggests that they should be highly efficient ways of combating chronic hepatitis B.

One critical hurdle to such developments has been the restriction of HBV infection to people and other higher primates, restricting the rapid development of new drugs in animal models. The development of humanised transgenic mice, available with and without a human immune system, will hopefully permit much faster experimental development^51,52^. This model and others will be useful for the evaluation of any novel therapeutic strategies being developed.

## Methods

### Patient data

Our study uses patient data comprising 6 patients whose their viral loads were recorded during the acute stage of infection and are reported in Ciupe *et al*.^5,8^, Webster *et al.*^28^ and Whalley *et al.*^53^. Patients 1, 2, 4, and 6 are female with an age range of 37 to 71. The data are published in the Supporting Information of Ciupe *et al.*^5^.

### Parameter estimation

It has been estimated that the total number of liver cells in an adult is equal to $2\times 10^{11}$ and only 60% of them are hepatocytes^8,23,54,55^. We assume that there are 3 liters of serum in an adult, containing complete and incomplete particles^24^. Therefore, $\lambda /d=0.6\times 2\times 10^{11}/3000=4\times 10^7 \text{ cells/ml }$. The death rate of target cells is estimated to be $0.01 \text{ day}^{-1}$^20,25^, so $\lambda =4\times 10^5$. As in unvaccinated patients with positive HBV-DNA test the antibody level is under $10 \text{ mIU/ml }=8.5\times 10^{-4} \text{ mg/ml }=3.4\times 10^{12} \text{ molecules/ml }$^26^, we assume that $A_m=3.4\times 10^{12} \text{ molecules/ml }$. For parameters $\phi$ (the level of infected cells at which growth is half-maximal) and $\xi$ (the rate at which functional effector cells are lost due to exhaustion) various fixed values have been determined previously^17,18^. We thus set $\phi =10^6 \text{ per } \text{ ml }$ and $\xi =1 \text{ day}^{-1}$. These values provide a good fit to the viral load data. All parameter values adapted from the literature are presented in Table 1 together with a pointer to the reference they have been adapted from. The remaining parameters are estimated by fitting the model (Fig. 1) to the viral load data using a modified version of DKLAG6 for the numerical simulations of the model (Fig. 1) in Fortran (see www.radford.edu for more detail)^56^ and an implementation of the Nelder-Mead algorithm in Fortran (see people.sc.fsu.edu for more detail)^57^. More details about the method are provided in the supplementary materials online (see Supplementary Section S2 online).

## Supplementary Information

Supplementary Figures.

Supplementary Video 1.

Supplementary Video 2.

## Data Availability

All data generated or analysed during this study are included in this published article (and its Supplementary Information files).

## References

[1] Mackay P, Lees J, Murray K (1981). The conversion of hepatitis B core antigen synthesized in E coli into e antigen. J. Med. Virol..

[2] Murray, K., Stahl, S. & Ashton-Rickardt, P. G. Genetic engineering applied to the development of vaccines. *Philos. Trans. R. Soc. Lond. B Biol. Sci.* **324**, 461–476 (1989). 10.1098/rstb.1989.0060 2573084

[3] WHO. *Global Hepatitis Report* (World Health Organization, 2021).

[4] Hu J, Liu K (2017). Complete and incomplete hepatitis B virus particles: formation, function, and application. Viruses.

[5] Ciupe, S. M., Ribeiro, R. M. & Perelson, A. S. Antibody responses during hepatitis B viral infection. *PLoS Comput. Biol.* **10**, e1003730 (2014). 10.1371/journal.pcbi.1003730 PMC4117427 25078553

[6] Guidotti LG (1999). Viral clearance without destruction of infected cells during acute HBV infection. Science.

[7] Abbas AK, Lichtman AH, Pillai S (2014). Cellular and molecular immunology.

[8] Ciupe SM, Ribeiro RM, Nelson PW, Dusheiko G, Perelson AS (2007). The role of cells refractory to productive infection in acute hepatitis B viral dynamics. Proc. Natl. Acad. Sci. USA.

[9] Ciupe SM, Ribeiro RM, Nelson PW, Perelson AS (2007). Modeling the mechanisms of acute hepatitis B virus infection. J. Theor. Biol..

[10] Fatehi Chenar, F., Kyrychko, Y. N. & Blyuss, K. B. Mathematical model of immune response to hepatitis B. *J. Theor. Biol.* **447**, 98–110 (2018). 10.1016/j.jtbi.2018.03.025 29574141

[11] Nelson PW, Gilchrist MA, Coombs D, Hyman JM, Perelson AS (2004). An age-structured model of HIV infection that allows for variations in the production rate of viral particles and the death rate of productively infected cells. Math. Biosci. Eng..

[12] Browne C (2016). Immune response in virus model structured by cell infection-age. Math. Biosci. Eng..

[13] Moskophidis D, Lechner F, Pircher H, Zinkernagel RM (1993). Virus persistence in acutely infected immunocompetent mice by exhaustion of antiviral cytotoxic effector T cells. Nature.

[14] Zajac AJ (1998). Viral immune evasion due to persistence of activated T cells without effector function. J. Exp. Med..

[15] Wherry EJ, Ahmed R (2004). Memory CD8 T-cell differentiation during viral infection. J. Virol..

[16] Revill PA (2019). A global scientific strategy to cure hepatitis B. Lancet Gastroenterol. Hepatol..

[17] Johnson PLF (2011). Vaccination alters the balance between protective immunity, exhaustion, escape, and death in chronic infections. J. Virol..

[18] Conway JM, Perelson AS (2015). Post-treatment control of HIV infection. Proc. Natl. Acad. Sci. USA.

[19] Fatehi F (2021). An intracellular model of hepatitis B viral infection: an in silico platform for comparing therapeutic strategies. Viruses.

[20] Guedj J (2013). Modeling shows that the NS5A inhibitor daclatasvir has two modes of action and yields a shorter estimate of the hepatitis C virus half-life. Proc. Natl. Acad. Sci. USA.

[21] Fatehi F, Kyrychko YN, Blyuss KB (2019). Time-delayed model of autoimmune dynamics. Math. Biosci. Eng..

[22] Jagadish, B. *Mathematical Modeling of T-cell Exhaustion and PD-1 Blockade in Chronic Infections*. Master’s thesis, University of California Irvine, the USA (2015).

[23] Goyal A, Ribeiro RM, Perelson AS (2017). The role of infected cell proliferation in the clearance of acute HBV infection in humans. Viruses.

[24] Murray JM, Goyal A (2015). In silico single cell dynamics of hepatitis B virus infection and clearance. J. Theor. Biol..

[25] Kitagawa K, Nakaoka S, Asai Y, Watashi K, Iwami S (2018). A PDE multiscale model of hepatitis C virus infection can be transformed to a system of ODEs. J. Theor. Biol..

[26] Guang Y, Yuzhong L, Hui L (2019). Establishment of an analysis model based on measurement of hepatitis B viral infection serum markers. BMC Infect. Dis..

[27] Wieland SF, Spangenberg HC, Thimme R, Purcell RH, Chisari FV (2004). Expansion and contraction of the hepatitis B virus transcriptional template in infected chimpanzees. Proc. Natl. Acad. Sci. USA.

[28] Webster GJM (2000). Incubation phase of acute hepatitis B in man: Dynamic of cellular immune mechanisms. Hepatology.

[29] Jack AD, Hall AJ, Maine N, Mendy M, Whittle HC (1999). What level of hepatitis B antibody is protective?. J. Infect. Dis..

[30] Chang M-L, Liaw Y-F (2014). Hepatitis B flares in chronic hepatitis B: Pathogenesis, natural course, and management. J. Hepatol..

[31] Perrillo RP (2001). Acute flares in chronic hepatitis B: The natural and unnatural history of an immunologically mediated liver disease. Gastroenterology.

[32] Lok AS, Zoulim F, Dusheiko G, Ghany MG (2017). Hepatitis B cure: From discovery to regulatory approval. J. Hepatol..

[33] Stray SJ (2005). A heteroaryldihydropyrimidine activates and can misdirect hepatitis B virus capsid assembly. Proc. Natl. Acad. Sci. USA.

[34] Stewart H (2016). Identification of novel RNA secondary structures within the hepatitis C virus genome reveals a cooperative involvement in genome packaging. Sci. Rep..

[35] Patel N (2017). HBV RNA pre-genome encodes specific motifs that mediate interactions with the viral core protein that promote nucleocapsid assembly. Nat. Microbiol..

[36] Twarock R, Stockley PG (2019). RNA-mediated virus assembly: Mechanisms and consequences for viral evolution and therapy. Ann. Rev. Biophys..

[37] Zoulim F (2017). Safety, tolerability, pharmacokinetics and antiviral activity of JNJ-56136379, a novel HBV capsid assembly modulator, in non-cirrhotic, treatment-naive subjects with chronic hepatitis B. In Hepatology.

[38] Lahlali T (2018). Novel potent capsid assembly modulators regulate multiple steps of the hepatitis B virus life cycle. Antimicrob. Agents Chemother..

[39] Zoulim F (2020). JNJ-56136379, an HBV capsid assembly modulator, is well-tolerated and has antiviral activity in a phase 1 study of patients with chronic infection. Gastroenterology.

[40] Reinharz, V. *et al.* Understanding hepatitis B virus dynamics and the antiviral effect of interferon-$$\alpha$$ treatment in humanized chimeric mice. *J. Virol.* **95**, e0049220 (2021). 10.1128/JVI.00492-20 PMC8223956 33910953

[41] Perelson AS, Ribeiro RM (2013). Modeling the within-host dynamics of HIV infection. BMC Biol..

[42] Czuppon, P. *et al.* Success of prophylactic antiviral therapy for SARS-CoV-2: Predicted critical efficacies and impact of different drug-specific mechanisms of action. *PLoS Comput. Biol.* **17**, e1008752 (2021). 10.1371/journal.pcbi.1008752 PMC7951973 33647008

[43] Gonçalves A (2020). Timing of antiviral treatment initiation is critical to reduce SARS-CoV-2 viral load. CPT Pharmacomet. Syst. Pharmacol..

[44] Iwanami, S. *et al.* Detection of significant antiviral drug effects on COVID-19 with reasonable sample sizes in randomized controlled trials: A modeling study. *PLoS Med.* **18**, e1003660 (2021). 10.1371/journal.pmed.1003660 PMC8259968 34228712

[45] Perelson AS, Ke R (2021). Mechanistic modeling of SARS-CoV-2 and other infectious diseases and the effects of therapeutics. Clin. Pharmacol. Ther..

[46] Guo, F. *et al.* HBV core protein allosteric modulators differentially alter cccDNA biosynthesis from de novo infection and intracellular amplification pathways. *PLoS Pathog.* **13**, e1006658 (2017). 10.1371/journal.ppat.1006658 PMC5629035 28945802

[47] Dahari H, Shudo E, Ribeiro RM, Perelson AS (2009). Modeling complex decay profiles of hepatitis B virus during antiviral therapy. Hepatology.

[48] Gao Y, Zhang T-Y, Yuan Q, Xia N-S (2017). Antibody-mediated immunotherapy against chronic hepatitis B virus infection. Hum. Vaccin. Immunother..

[49] Cerino A (2019). Human monoclonal antibodies as adjuvant treatment of chronic hepatitis B virus infection. Front. Immunol..

[50] Alexopoulou A, Vasilieva L, Karayiannis P (2020). New approaches to the treatment of chronic hepatitis B. J. Clin. Med..

[51] Dusséaux M (2017). Viral load affects the immune response to HBV in mice with humanized immune system and liver. Gastroenterology.

[52] Li Y (2018). A human immune system mouse model with robust lymph node development. Nat. Methods.

[53] Whalley SA (2001). Kinetics of acute hepatitis B virus infection in humans. J. Exp. Med..

[54] Michalopoulos GK (2007). Liver regeneration. J. Cell. Physiol..

[55] Kmiec Z (2001). Cooperation of liver cells in health and disease. Adv. Anat. Embryol. Cell Biol..

[56] Thompson S, Shampine LF (2006). A friendly fortran DDE solver. Appl. Numer. Math..

[57] O’Neill R (1971). Algorithm AS 47: Function minimization using a simplex procedure. J. R. Stat. Soc. C-Appl..

