# Supplementary material for: An age-structured model of hepatitis B viral infection highlights the potential of different therapeutic strategies
Source: Sci Rep. 2022 Jan 24;12:1252. doi: 10.1038/s41598-021-04022-z (PMC8786976; doi:10.1038/s41598-021-04022-z)
Supplement: Supplementary file 1 — Supplementary Figures. [file 41598_2021_4022_MOESM1_ESM.pdf]

Supplementary information, figures and tables

This file contains the supplementary material to support the manuscript: "*An age-structured model of hepatitis B viral infection highlights the potential of different therapeutic strategies*", submitted to the Scientific Reports journal.

Farzad Fatehi[1,2], Richard J Bingham[1,2,3], Peter G Stockley[4], and Reidun Twarock[1,2,3,*]

[1] *York Cross-disciplinary Centre for Systems Analysis, University of York, York YO10 5GE, UK*
[2] *Department of Mathematics, University of York, York YO10 5DD, UK*
[3] *Department of Biology, University of York, York YO10 5NG, UK*
[4] *Astbury Centre for Structural Molecular Biology, University of Leeds, Leeds LS2 9JT, UK*
[*] *Corresponding Author: rt507@york.ac.uk*

Supplementary Figures

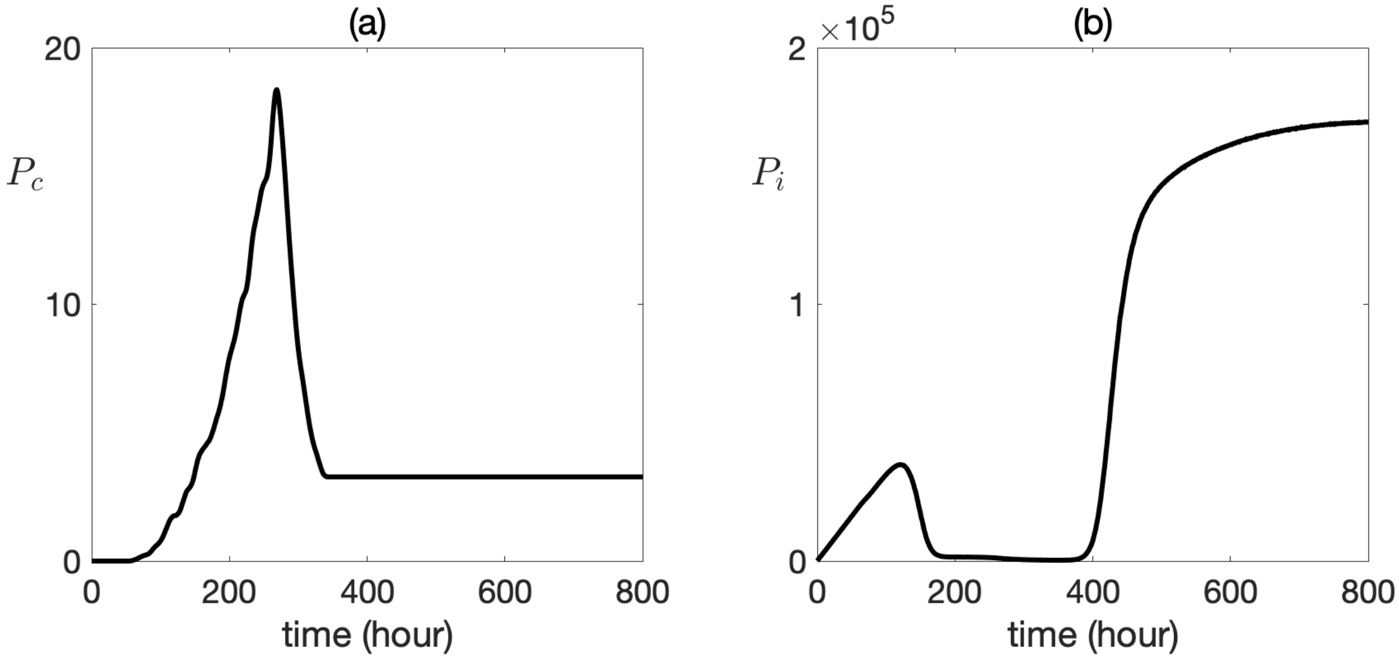

Figure S1: The production profiles of particles. (**a**) and (**b**) indicate the release profiles of complete ($P_c(.)$) and incomplete ($P_i(.)$) particles, respectively.

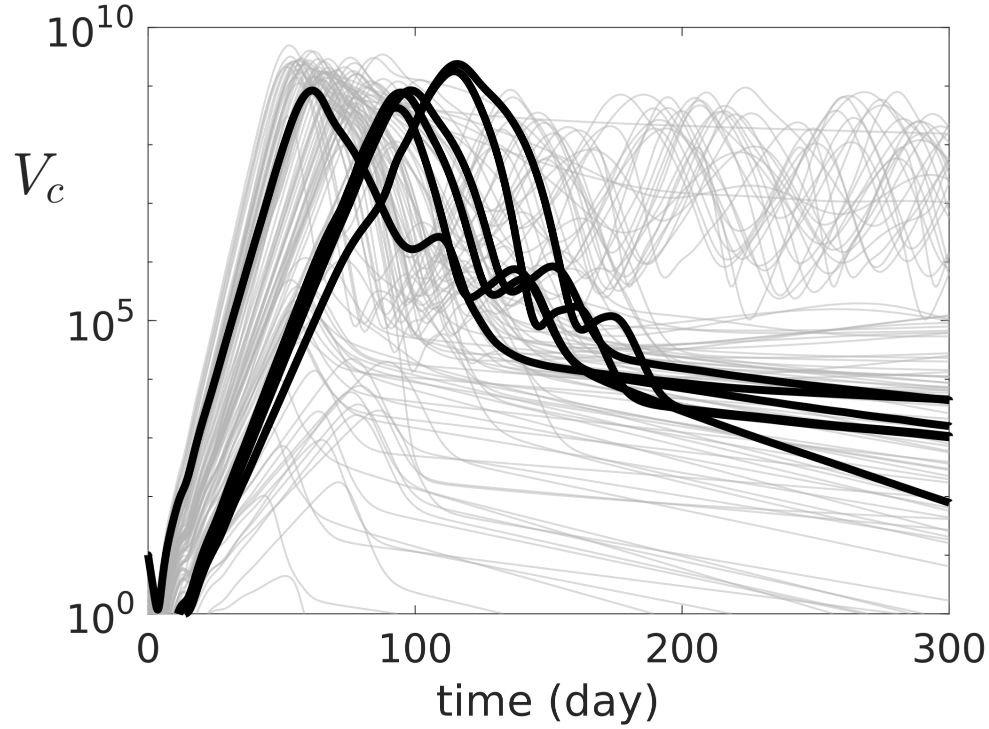

Figure S2: 100 numerical simulations of the model (Fig. 1) using 100 parameter sets that are inferred from the fitting of parameter values form the six patients to a Gaussian distribution. The gray curves show the 100 simulations while the six black curves indicate the fitted curves to patient data. 60% of simulations leads to an acute infection, 25% of simulations show a periodic solution while 15% of them leads to a stable chronic infection steady state with a low level of free viruses (around 10^5 virion per ml).

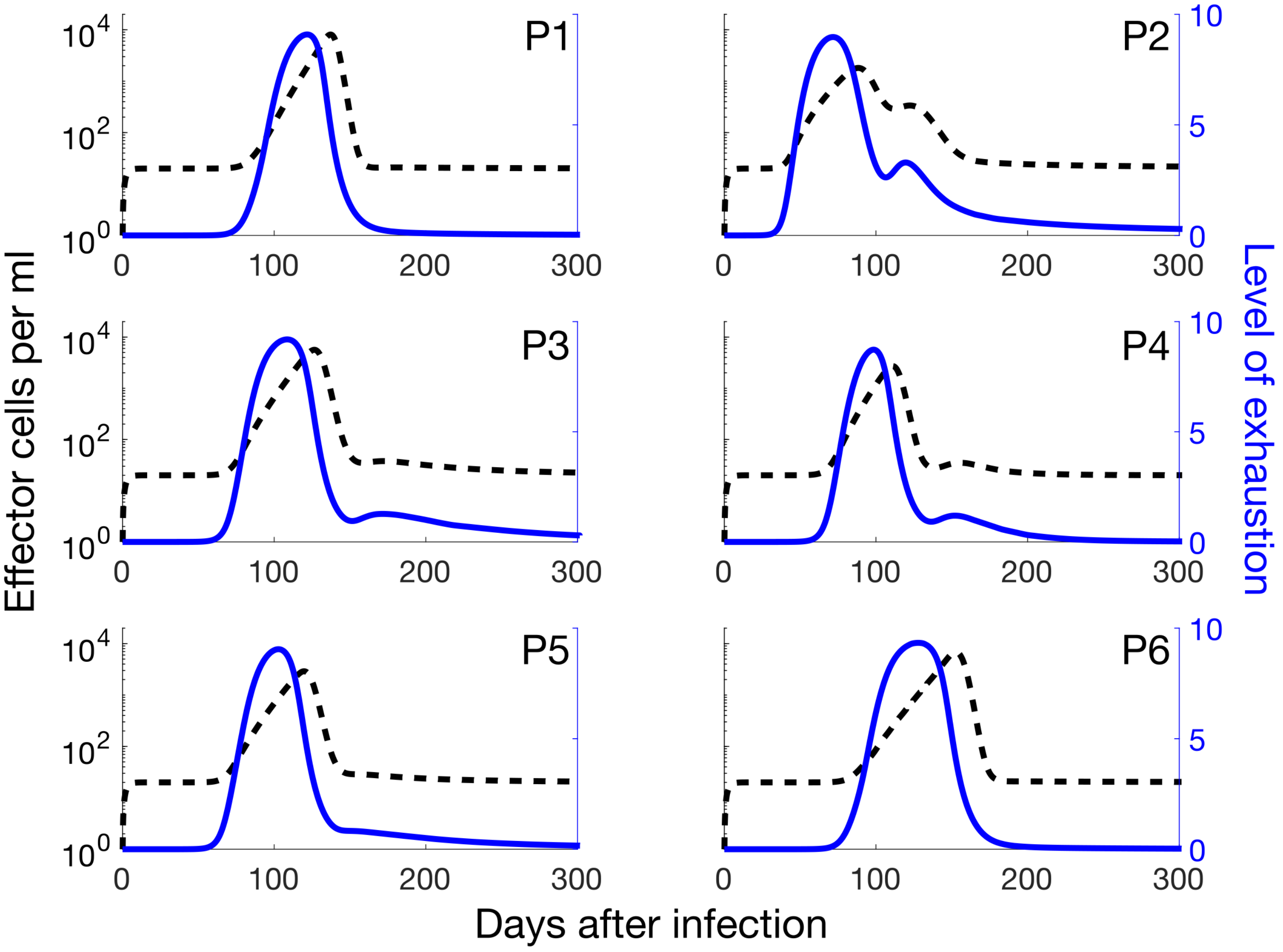

Figure S3: The level of T cells exhaustion declines before the peak of effector cells in acute HBV infections. Blue lines indicate the level of exhaustion and black dashed lines show the dynamics of effector cells.

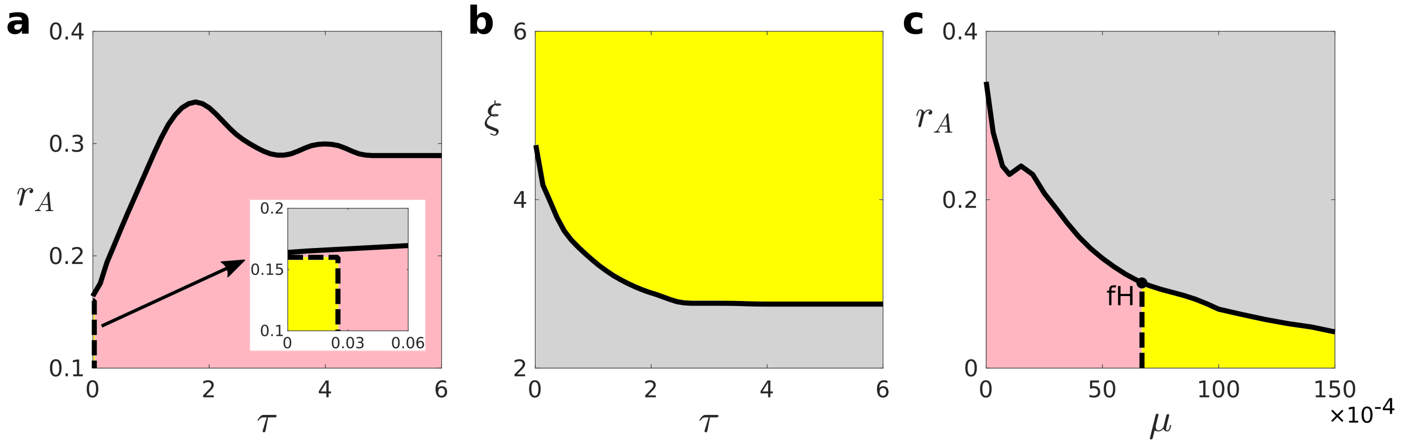

Figure S4: Stability analysis of the disease-free and chronic infection states. The parameters used are the median values from Table 2. In (**a**), (**b**) and (**c**) the gray and yellow areas indicate the regions where the disease-free and chronic infection steady states are stable, respectively. Pink is the region where the system shows a stable periodic solution around a chronic infection steady state. Solid and dashed lines indicate the boundaries of the steady-state and Hopf bifurcation, respectively, and "fH" shows the location of the fold-Hopf bifurcation.

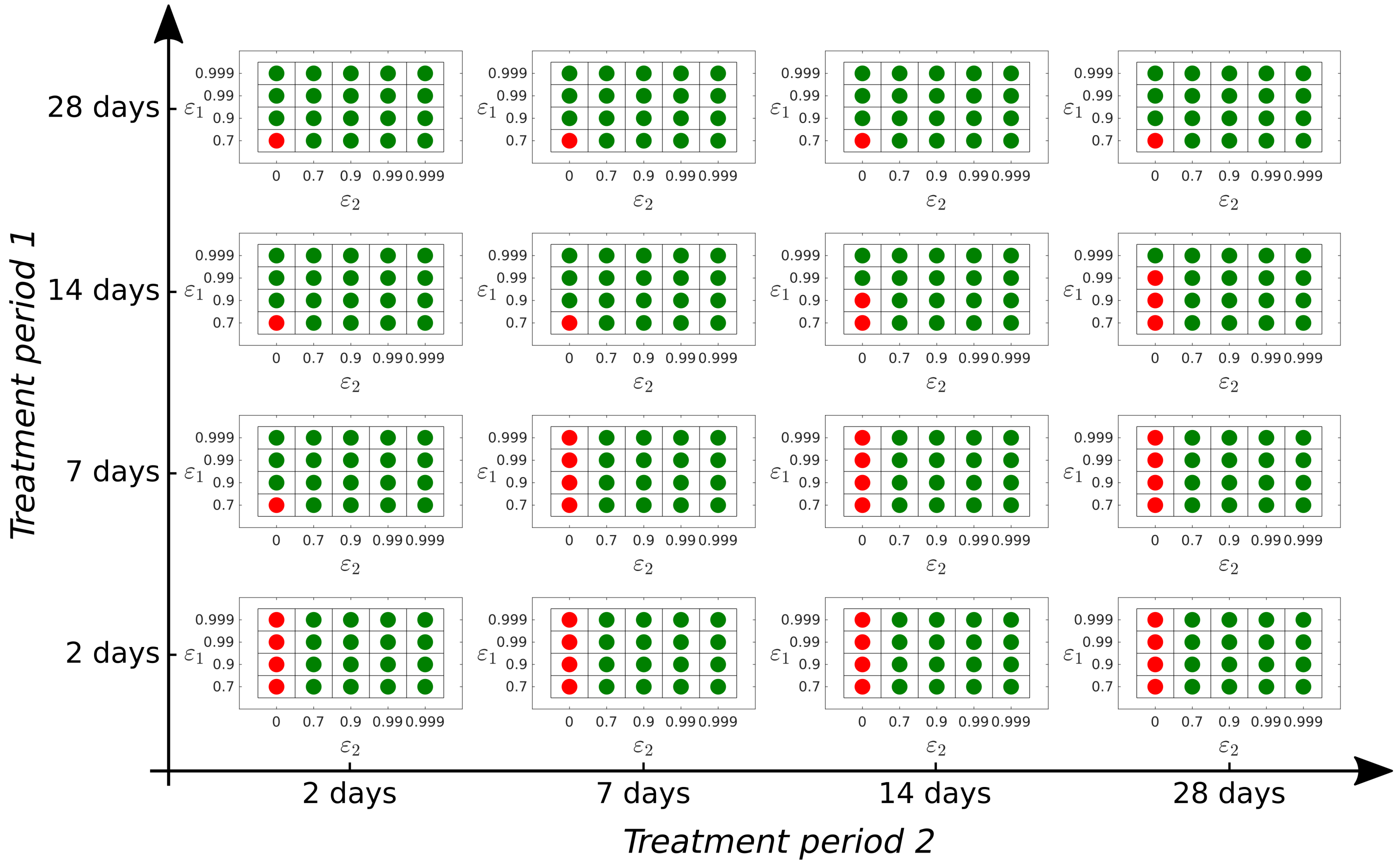

Figure S5: Periodic treatment starting at 130 dpi with alternating treatments of different efficacy. Parameter values are as in Fig. 5c to enable comparison with the continuous treatment option. X and Y axes indicate the duration of each treatment period, and ε_1 and ε_2 the total efficacy of the treatment in each period. Green and red dots indicate successful (clearance of infection) and unsuccessful (viral rebound) outcomes, respectively, at the end of 48 weeks (the recommended time for HBV therapy) of alternating treatment.

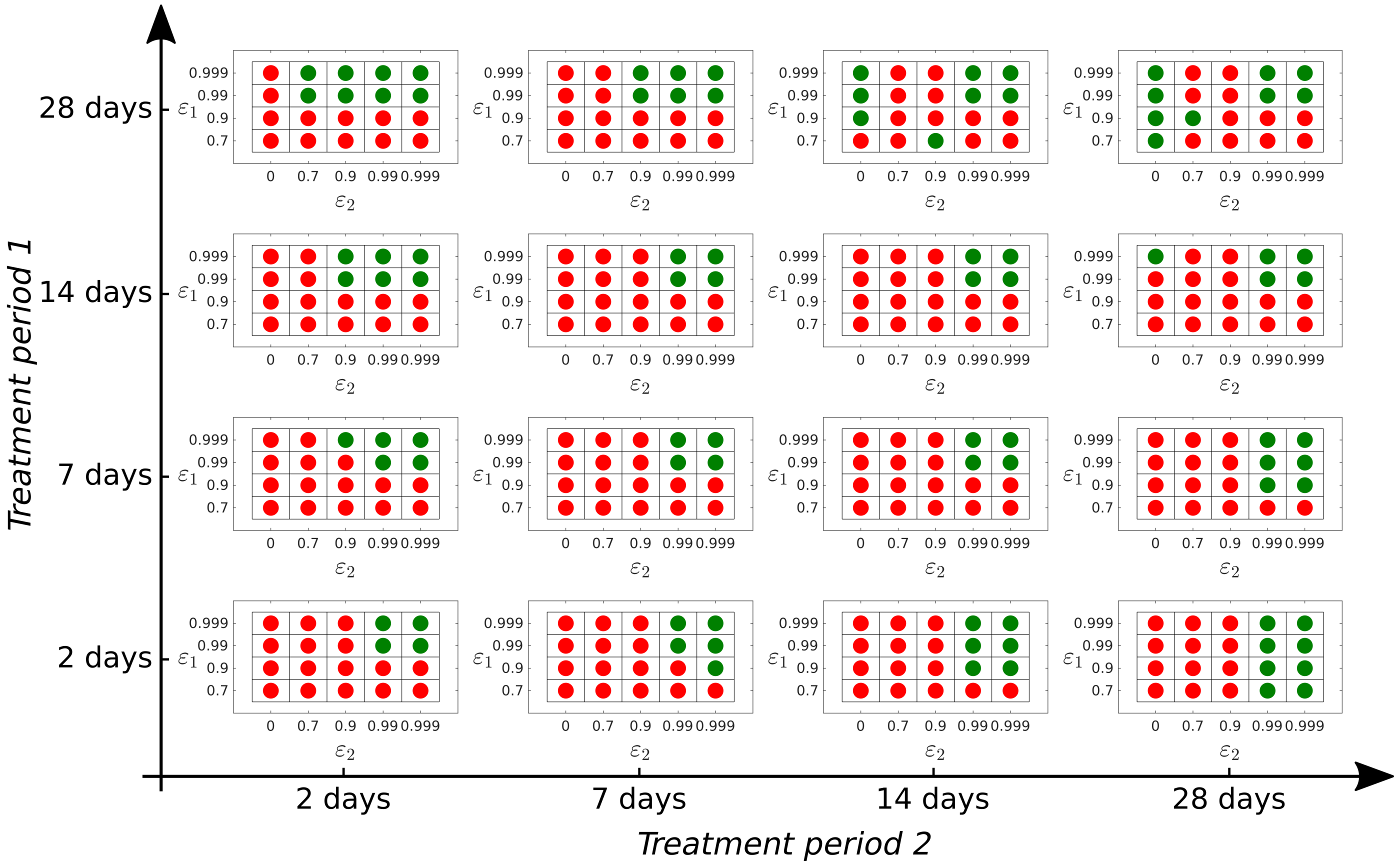

Figure S6: Periodic treatment starting at 150 dpi with alternating treatments of different efficacy. Parameter values are as in Fig. 5c to enable comparison with the continuous treatment option. X and Y axes indicate the duration of each treatment period, and ε_1 and ε_2 the total efficacy of the treatment in each period. Green and red dots indicate successful (clearance of infection) and unsuccessful (viral rebound) outcomes, respectively, at the end of 48 weeks (the recommended time for HBV therapy) of alternating treatment.

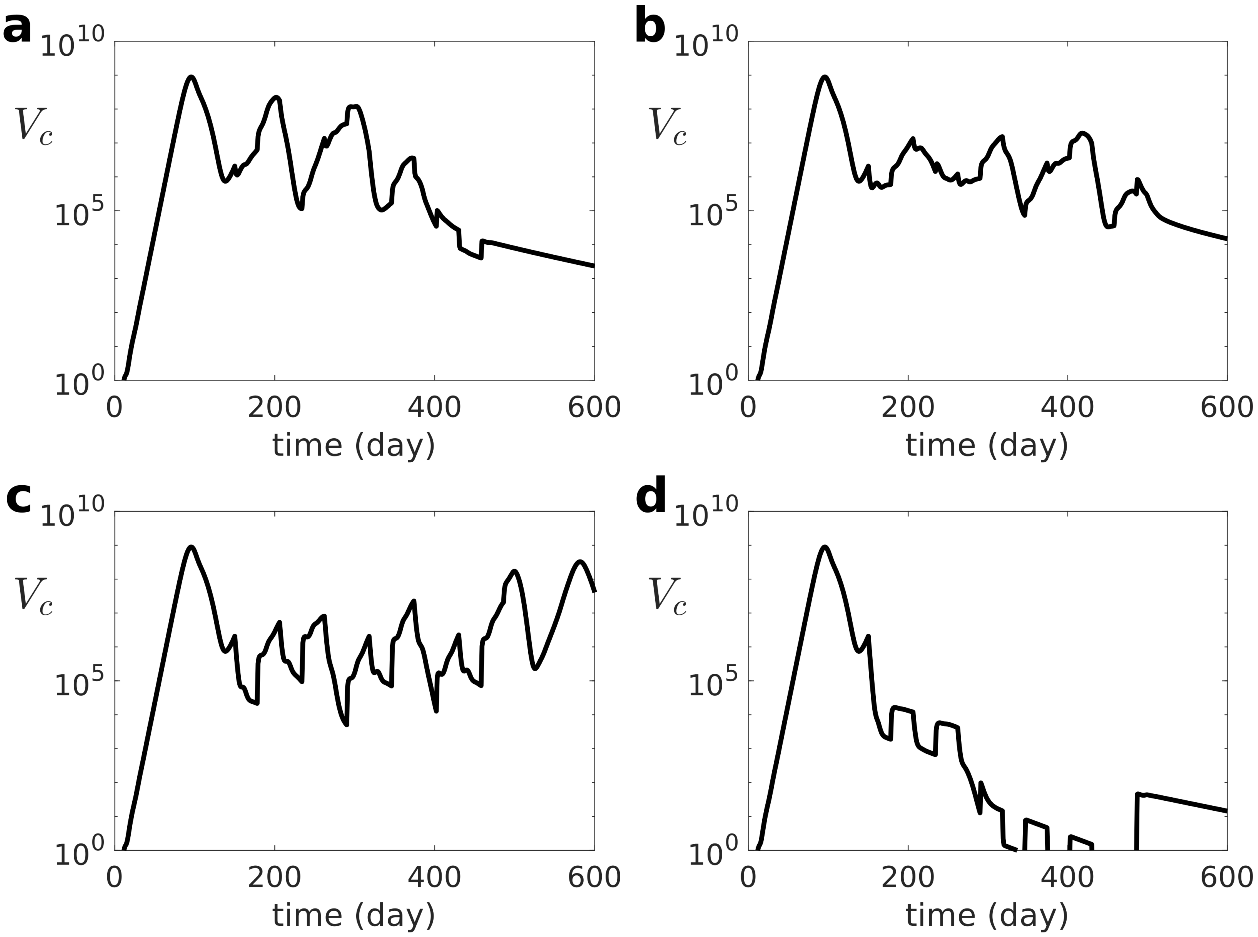

Figure S7: Viral load curves for alternating periodic treatments of 4 weeks for treatment starts at 150 dpi (parameter values as in Fig. 5c). The respective efficacies in treatment periods 1 and 2 are: (**a**) $\varepsilon_1 = 0.7$ and $\varepsilon_2 = 0$ (i.e., no treatment in period 2); (**b**) $\varepsilon_1 = 0.9$ and $\varepsilon_2 = 0.7$; (**c**) $\varepsilon_1 = 0.99$ and $\varepsilon_2 = 0.7$; and (**d**) $\varepsilon_1 = 0.999$ and $\varepsilon_2 = 0.99$.

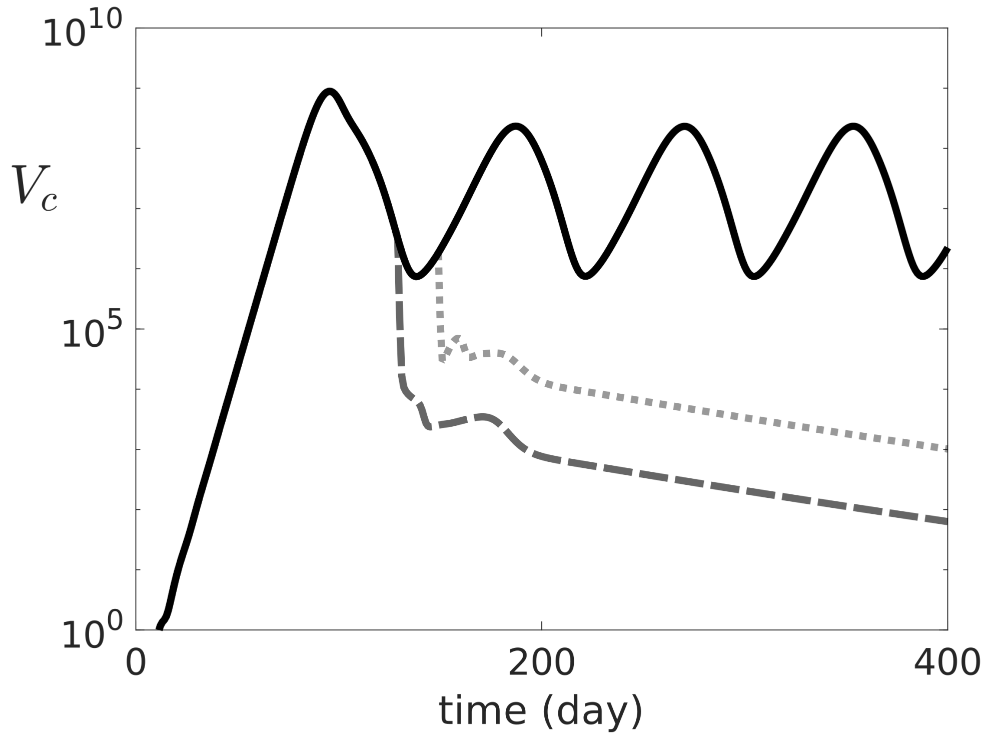

Figure S8: Start of antibody therapy is independent of the viral dynamics in both declining and increasing phases leads to the infection removal. The parameters used are the median values from Table 2 with $\tilde{A}_m = 3.4 \times 10^{12}$ in the model (S1). The dashed and dotted lines, represent the effects of treatments on viral load, for treatment starts 130 and 150 dpi, respectively.

Supplementary Videos captions

Supplementary Video S1: The minimal total efficacy (ϵ_{tot}) that is required for an antiviral therapy to clear the infection following a 48 weeks therapy starting at various times between 50 dpi and 160 dpi. The white area indicates a stable disease-free state. The red solid curves indicate the onset of the steady-state bifurcation, whereas the red dotted lines of the Hopf bifurcation of the chronic state. The black hatched area indicates the region where an efficacy of $\leq 99.99\%$ is ineffective.
Supplementary Video S2: The minimal efficacy (η) that is required for exhaustion therapy to clear the infection following a 48 weeks therapy starting at various times between 50 dpi and 160 dpi. The white area indicates a stable disease-free state. The red solid curves indicate the onset of the steady-state bifurcation, whereas the red dotted lines of the Hopf bifurcation of the chronic state. The black hatched area indicates the region where an efficacy of $\leq 99.99\%$ is ineffective.

S1 Modelling the effect of monoclonal antibody therapy

Monoclonal anti-HBsAg antibody drugs are currently investigated in clinical trials [1, 2, 3]. The impact of this treatment option is modelled as an influx antibodies into the system. The new equations take

the following form

$$
\begin{aligned}
\frac{dV_c}{dt} &= \rho_1 \int_0^\infty P_c(a) I(a,t) da - d_c V_c - k_f A V_c + k_b X_c + \theta(t - t_A)(-k_f \tilde{A} V_c + k_b \tilde{X}_c), \\
\frac{dV_i}{dt} &= \rho_2 \int_0^\infty P_i(a) I(a,t) da - d_i V_i - k_f A V_i + k_b X_i + \theta(t - t_A)(-k_f \tilde{A} V_i + k_b \tilde{X}_i), \\
\frac{d\tilde{A}}{dt} &= \theta(t - t_A)(-d_A \tilde{A} - k_f \tilde{A}(V_c + V_i) + k_b(\tilde{X}_c + \tilde{X}_i)), \\
\frac{d\tilde{X}_c}{dt} &= \theta(t - t_A)(k_f \tilde{A} V_c - k_b \tilde{X}_c - d_x \tilde{X}_c), \\
\frac{d\tilde{X}_i}{dt} &= \theta(t - t_A)(k_f \tilde{A} V_i - k_b \tilde{X}_i - d_x \tilde{X}_i),
\end{aligned}
\tag{S1}
$$

where $\tilde{A}(t) = 0$ for $t < t_A$ and $\tilde{A}(t_A) = \tilde{A}_m$, with $\tilde{A}_m$ representing the number of antibodies per ml that are administered as treatment. t_A denotes the time at which the treatment is started, and $\theta(.)$ is the Heaviside function.

S2 Parameter estimation

As mentioned in Methods, to estimate the parameter values for the model (Fig. 1), we used an implementation of the Nelder-Mead algorithm in Fortran (see people.sc.fsu.edu for more detail) [4] to minimise the following function:

$$
f(\theta) = \sum_{i=1}^{m} (\log(V_c(t_i, \theta)) - \log(\overline{V}(t_i))),
$$

where m is the number of measured data points, $\theta = [\beta, \mu, \delta, \rho_1, \rho_2, p_A, r_A, \alpha, \tau]$ is the vector of parameters, $V_c(t_i, \theta)$ is the level of complete virions, at time t_i with parameter values θ, generated by the model and $\overline{V}(t_i))$ is the level of measured free virus at time t_i. For each set of parameters, we generate a numerical solution of the model (Fig. 1), $V_c(t_i, \theta)$, via a modified version of DKLAG6 in Fortran (see www.radford.edu for more detail) [5]. When $f(\theta)$ is minimised the best fit occurs.

References

[1] Gao, Y., Zhang, T.-Y., Yuan, Q. & Xia, N.-S. Antibody-mediated immunotherapy against chronic hepatitis B virus infection. *Hum. Vaccin. Immunother.* **13**, 1768–1773 (2017).

[2] Cerino, A. *et al.* Human monoclonal antibodies as adjuvant treatment of chronic hepatitis B virus infection. *Front. Immunol.* **10**, 2290 (2019).

[3] Alexopoulou, A., Vasilieva, L. & Karayiannis, P. New approaches to the treatment of chronic hepatitis B. *J. Clin. Med.* **9**, 3187 (2020).

[4] O'Neill, R. Algorithm AS 47: function minimization using a simplex procedure. *J. R. Stat. Soc. C-Appl.* **20**, 338–345 (1971).

[5] Thompson, S. & Shampine, L. F. A friendly fortran DDE solver. *Appl. Numer. Math.* **56**, 503–516 (2006).
